# A de novo* ARIH2* gene mutation was detected in a patient with autism spectrum disorders and intellectual disability

**DOI:** 10.1038/s41598-024-66475-2

**Published:** 2024-07-09

**Authors:** Mirella Vinci, Simone Treccarichi, Rosanna Galati Rando, Antonino Musumeci, Valeria Todaro, Concetta Federico, Salvatore Saccone, Maurizio Elia, Francesco Calì

**Affiliations:** 1 Oasi Research Institute-IRCCS, 94018 Troina, Italy; 2 Department of Medical and Surgical Sciences and Advanced Technologies “G.F. Ingrassia”, University of Catania, Catania, Italy; 3 Department of Biological, Geological and Environmental Sciences, University of Catania, Via Androne 81, 95124 Catania, Italy

**Keywords:** Ubiquitination, Whole exome sequencing, Autism spectrum disorder, E3 ubiquitin-protein ligase, Splicing region, Autosomal dominant inheritance model, Mutation, Next-generation sequencing, Cellular neuroscience, Neurological disorders

## Abstract

E3 ubiquitin protein ligase encoded by *ARIH2* gene catalyses the ubiquitination of target proteins and plays a crucial role in posttranslational modifications across various cellular processes. As prior documented, mutations in genes involved in the ubiquitination process are often associated with autism spectrum disorder (ASD) and/or intellectual disability (ID). In the current study, a de novo heterozygous mutation was identified in the splicing intronic region adjacent to the last exon of the *ARIH2* gene using whole exome sequencing (WES). We hypothesize that this mutation, found in an ASD/ID patient, disrupts the protein Ariadne domain which is involved in the autoinhibition of ARIH2 enzyme. Predictive analyses elucidated the implications of the novel mutation in the splicing process and confirmed its autosomal dominant inheritance model. Nevertheless, we cannot exclude the possibility that other genetic factors, undetectable by WES, such as mutations in non-coding regions and polygenic risk in inter-allelic complementation, may contribute to the patient's phenotype. This work aims to suggest potential relationship between the detected mutation in *ARIH2* gene and both ASD and ID, even though functional studies combined with new sequencing approaches will be necessary to validate this hypothesis.

## Introduction

Genomic variants located outside of the canonical splicing sites (± 2) can lead to abnormal mRNA splicing and are classified as non-canonical splicing variants (NCSVs). The clinical implications of NCSVs in neurodevelopmental disorders (NDDs) remain largely unexplored. Recent studies have underscored the role of intronic variants in disrupting critical neurodevelopmental pathways, suggesting their potential involvement in conditions such as autism spectrum disorder (ASD) and intellectual disability (ID)^1–3^. Among the potentially affected pathways, those regulated by ubiquitination enzymatic activity may be linked to the onset of neurodevelopmental disorders^4^. However, it is important to note that the genetic mechanisms linking non-coding DNA regions to ubiquitination and their impact on neurodevelopmental disorders remain unknown. Detecting smaller, often multiple copy number variants (CNVs) affecting putative regulatory elements may provide insights into additional risks associated with simplex neurodevelopmental disorders, including autism^5,6^.

Protein ubiquitination is a posttranslational modification encompassing various fundamental cellular processes^7^. Currently, about 600–700 ubiquitin ligase genes have been detected, representing ~ 5% of the human genes. Protein ubiquitination involves a sophisticated enzymatic cascade comprising ubiquitin-activating (E1), ubiquitin-conjugating (E2), and ubiquitin ligase (E3) enzymes^8^. E1 enzymes initiate the ubiquitination process by activating ubiquitin through ATP-dependent adenylation. The activated ubiquitin is subsequently transferred to a E2 enzyme, which acts as an intermediary delivering the ubiquitin to a E3 ligase^9^. This highly coordinated process ensures the precise attachment of ubiquitin molecules to target proteins, orchestrating a diverse array of cellular responses and functions^10,11^. E2-E3 complexes play a pivotal role in this process, orchestrating the attachment of ubiquitin chains to proteins. The fate of ubiquitinated proteins is determined by the type of ubiquitin chain formed. This intricate regulatory system highlights the multifaceted impact of protein ubiquitination on cellular function.

The ubiquitin–proteasome system (UPS) regulates cellular protein levels crucial for neuronal growth and function, impacting key processes in the nervous system^12^. Mutations in E3 ligase genes are associated with various neurological conditions^13^. In fact, ubiquitination is intricately linked to autism spectrum disorder (ASD), neurodevelopmental impairments, and intellectual disability (ID)^9^. Understanding the UPS's role in these disorders provides insights into their molecular mechanisms, influencing neural development and signalling pathways like WNT, mTOR, and TGFβ. UPS activity directly influences axonal and dendritic development, synaptic maturation, and pruning^14–16^. E3 ligase genes are implicated in ASD, schizophrenia, ID, epilepsy, Parkinson’s disease, and Angelman syndrome, highlighting their broad impact on neurological health^9,13,17–20^.

E3 enzymes are categorized into three distinct groups: the really interesting new gene (RING) group, the homologous to E6-AP carboxyl terminus (HECT), and the RING-between-RING (RBR) E3 family. RING ligases operate by directly transferring ubiquitin from E2 enzymes to the substrate through substrate hydrolysis. In contrast, HECT and RBR E3 ligases follow a distinct mechanism. They catalyse ubiquitin transfer to a lysine residue on the E3 ligase itself before subsequently transferring it to the target substrate^9^. Within this context, Ariadne RBR E3 ubiquitin protein ligase 2 (ARIH2) is a RBR family member, which exhibits unique E3 ligase activity, interacting with Ubiquitin-conjugating enzyme E2 L3 (UBE2L3) and functioning similarly to HECT-type E3 enzymes. Particularly, ARIH2 catalyzes ubiquitination of target proteins together with UBE2L3^21,22^. These enzymes bind E2s through the first RING-type zinc finger, requiring an obligate trans-thiolation step for ubiquitin transfer involving a conserved cysteine residue in the second RING-type zinc finger^23^.

The E3 ubiquitin protein ligase enzyme encoded by *ARIH2* gene comprises 8 highly conserved domains, encompassing three zinc finger type domains: RING-type 1, IBR-type and RING-type 2^24^. As previously documented, E3 ubiquitin protein ligase is autoinhibited by the Ariadne domain, which masks the active site within the second RING-type zinc finger domain, thereby inhibiting E3 activity^19^. Within this context, ARIH2 ubiquitinates substrates of E3 ubiquitin ligase complexes composed of the neddylated forms of Cullin 5 and RING-box protein 2 (neddylated CUL5-RBX2 E3s). Structural analyses reveal ARIH2's distinctive autoinhibition and activation upon association with neddylated CUL5-RBX2, interacting with CUL5 and NEDD8^25,26^.

*ARIH2* gene is intricately involved in embryonic development through its direct interaction with Hedgehog signalling and myelopoiesis process^27,28^. In particular, it directly interplays with Hedgehog signalling through smoothened ubiquitylation and endoplasmic reticulum—associated degradation^28^. According to documented evidence, *ARIH2* gene is ubiquitously expressed, showing its highest expression levels in granulocytes^29^. Additionally, *ARIH2* shows high expression patterns in several brain tissues, including amygdala, basal ganglion, cerebellar hemispheres, cerebellar vermis, and cerebral cortex (The Human Protein Atlas). Disorders in these brain regions were previously described as potentially involved to neurodevelopmental diseases including ASD and ID^30–32^. *ARIH2* is a paralog of *ARIH1* gene, and both participate in DNA repair against genotoxic stress and potentially mediate mRNA translation arrest due to its shared Ariadne domain^33^. The human homolog of Ariadne and counterparts in Drosophila and *Caenorhabditis elegans* are implicated in regulating translation, cellular proliferation, and developmental processes^34,35^.

The objective of this study was to verify a new potential relationship between ASD and ID and a de novo novel mutation in an intronic splicing region adjacent to the last exon of *ARIH2* gene. This report contributes to further reinforce the knowledge of the genetic basis related to ASD and ID, suggesting the possible involvement of the Ubiquitin E3 ligase pathway in the manifestation of these neurodevelopmental disorders. Additionally, it raises the possibility that mutations in intronic regions, which are often undetectable by WES, may significantly contribute to the patient's phenotype.

## Results

### Clinical report

The 20-year-old girl was the second daughter of healthy, non-consanguineous parents. She was born at term; no complications occurred during pregnancy. The birth weight registered was 3100 g (z-score: − 0.3), the length was 52 cm (z-score: 1.5) At birth, she presented jaundice, that did not require phototherapy. She presented psychomotor delay: autonomous walking occurred at 20 months of age, first words at 18–20 months of age, sphincter control at the age of 5. When she was 2 years old, the first relational abnormalities were noted: she tended to hide behind her mother, when called, she didn’t promptly answer. At the kindergarten, at the age of 3, she was very shy, spoke softly, she used to play putting the dolls one behind the other, sometimes she launched them. Subsequently, she started to manifest behavioral problems: anxiety reactions, mood instability, repetitiveness, a strong addiction for the mum, episodes of loss of control, hand stereotypies, nocturnal hyperphagia. She was diagnosed of ASD at the age of 10 and treated with quetiapine, risperidone, lamotrigine, cariprazine, without benefit. Brain MRI was unremarkable.

In the 7 months preceding the admission to our department, at the age of 19, she presented frequent episodes (more times per week), of feeling of anxiety, cough, vomiting, headache, lasting for many hours. A slowing in movements and a delay before starting to speak were also noted at that time.

At the age of 20, the clinical phenotype was characterized by downwards-pointing eyelid fissures, posterior auricles, kyphoscoliosis, hand-stereotypies, accentuated during speech, bilateral postural tremor and tendency to keep the hands flexed during walking. Her weight was 54 kg (25–50 centile), her height was 151.5 cm (3–10 centile), her BMI was 23.68 kg/m^2^, head circumference was 55 cm (50–75 centile). At that time, she was treated with haloperidol 2 mg/die, melatonin 2 mg/die. In order to manage her behavioral symptoms, olanzapine was added to her therapy, at the dosage of 10 mg/die, with improvement. The EEG showed sharp-waves and spikes over the temporal regions of the left hemisphere, with tendency to contralateral propagation. Furthermore, magnetic resonance imaging (MRI) of the brain displayed normal results. From a psychological point of view, the diagnosis of ASD level 2 was confirmed, and a diagnosis of moderate ID was made. Comparative genome hybridization (CGH) array didn’t reveal any CNV alterations.

### Genetic analysis by next generation sequencing

**Figure 1:**
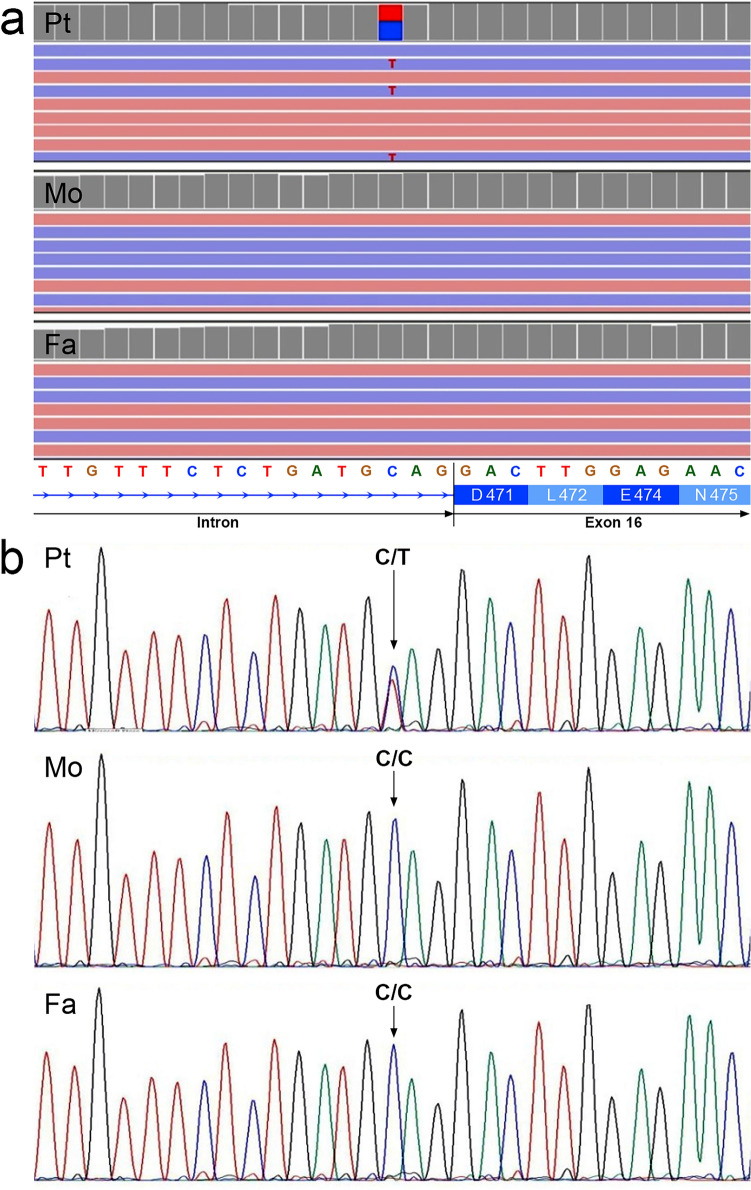
(**a**) Graphical representation of the heterozygous de novo variation identified within *ARIH2* gene. Images display data from the patient (Pt), the mother (Mo), and the father (Fa). The novel mutation, detected using the Integrative Genome Viewer (IGV) system, is illustrated. At the bottom is displayed the nucleotide sequence and the corresponding amino acids of the region. (**b**) The de novo mutation was confirmed through conventional Sanger sequencing in the patient (Pt), the mother (Mo), and the father (Fa).

Whole exome sequencing revealed the presence of a heterozygous de novo nucleotide variation at position c.1411-3C > T, within the last intron in the *ARIH2* gene (NM_006321) (Fig. 1).

Sanger sequencing on the parents confirmed the presence of this mutation only in the patient affected by ASD (Fig. 1). WES analysis unveiled no additional mutations directly or potentially associated with other known ASD and/or ID related genes. Furthermore, to confirm that the observed mutation can be considered a de novo mutation, short tandem repeat (STR) polymorphisms were analysed in the patient/mother/father subjects, confirming their parenthood.

**Figure 2:**
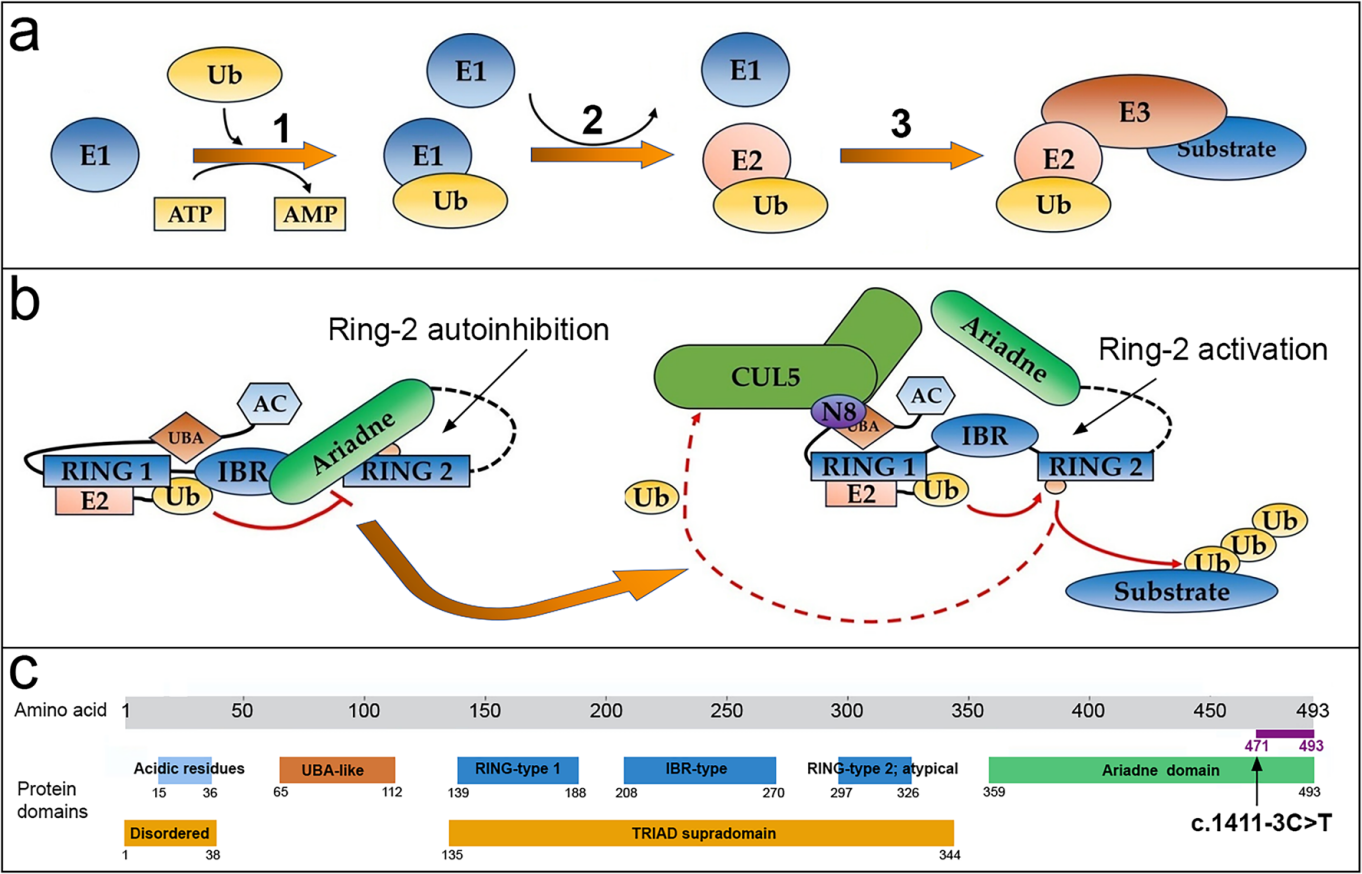
(**a**) Depiction of the ubiquitination mechanism. Specifically, this process involves E1, E2, and E3 enzymes. Precisely, E1 initiates the process by the ubiquitin activation (step 1). Subsequently, the activated ubiquitin is transferred to an E2 enzyme (step 2), which delivers it to an E3 ligase (step 3). This process ensures the targeted attachment of ubiquitin to a protein substrate, regulating various cellular functions. (**b**) Autoinhibition of the E3 ubiquitin protein ligase activity operated by the Ariadne domain. Specifically, this domain blocks the second RING-type zinc finger domain, thereby inhibiting E3 activity. Cullin-5 (CUL5) and NEDD8 (N8) are core components of the E3 ubiquitin-protein ligase complex which mediate the proteasomal degradation of target proteins^26^. The graphical representation is a modified version of a previous model related to ARIH1 (whose gene is paralog of ARIH2) having the same domains pattern organization: acidic residues (AC)-UBA-RING1-IBR-RING2-Ariadne^36^. (**c**) Graphical representation of the 8 highly conserved domains within the ARIH2 protein. The figure also highlights the specific position of the identified mutation (c.1411-3C > T), that likely affects the encoding region of the ARIH2 protein from amino acid 471, within the last portion of the Ariadne domain. The figure was modified from the Uniprot protein database.

The mutation is situated at -3 base pairs from the terminal exon, which encodes the final segment of the Ariadne domain (amino acids 359 to 493) as depicted in Fig. 2. Conversely, the nucleotide variation observed resulted in a change, from T to C, within the splicing region of donor site sequence, adjacent to the last exon. The causative pathogenetic effect of this specific variant was obtained on the assessment conducted using the ACMG criteria and, more specifically, three criteria for the classification were used, supporting (PP4), moderate (PM2), and strong (PS2) association.

### In silico* splicing prediction*

**Figure 3:**
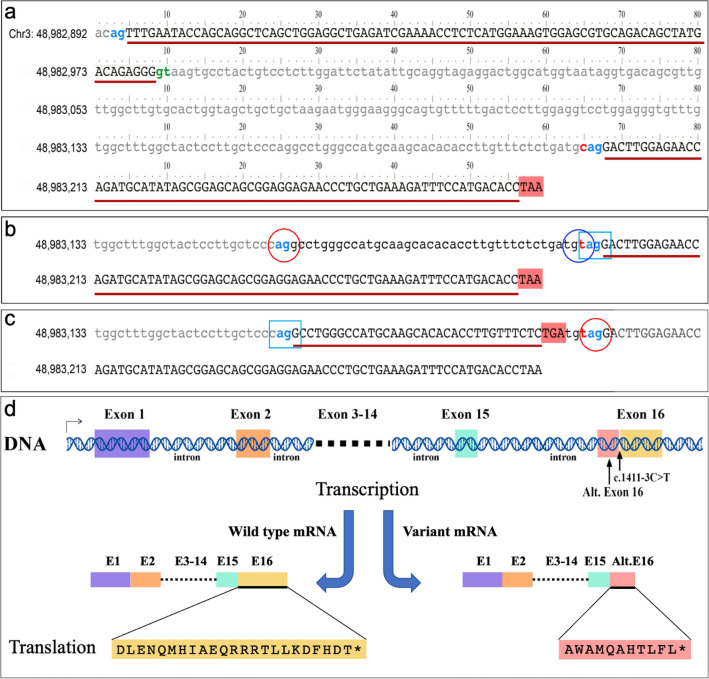
In silico splicing predictions related to the variant c.1411-3C > T in the *ARIH2* gene. (**a**) Genomic sequence of the exon 15 and 16, and the intron between them, of the *ARIH2* gene. Exon and intron sequences are in uppercase and lowercase letters, respectively. The red lines indicate the translated sequences. The “ag” blue light and the “gt” green nucleotides indicate the acceptor/donor splicing sites respectively. The TAA sequence in a red box indicate the stop codon. On the left, the genomic position, on the chromosome 3, of the first nucleotide on the same line, were indicated. The red “c” nucleotide indicated the site involved in the c.1411-3C > T variant. (**b**) Genomic sequence of the exon 16 and the last part of the intron with the c.1411-3C > T variant (red nucleotide) showing the “ag” (red circle) and the “gt” (blue circle) putative splicing sites. The blue light box indicates the acceptor splicing site determining the wild type mRNA formation. (**c**) Genomic sequence as in (**b**). The blue light box indicates the acceptor splicing site determining, if used, a different mRNA formation, which include a sequence with a stop codon (indicated by the red box) after 11 codons. Red circle indicates the “ag” splicing sites here not used. (**d**) Schematic representation of the two mRNA variants, and the relative amino acid codified, on the basis to the use of the two “ag” acceptor sites shown in “b”, and (**c**). Alt. Exon 16: alternative exon 16 present in the mRNA in the case the “ag” splicing site at position 48,983,157 is used. Exons, introns, and mRNA are not to scale.

*In-silico* splicing prediction analysis was performed considering the putative new donor/acceptor splicing sites identified close to the start of the exon 16 of the *ARIH2* gene (Fig. 3). The analysis taken in consideration the new putative donor site “gt”, directly obtained by the variant c.1411-3C > T, further to the putative acceptor site “ag” at the position 48,983,157 (Fig. 3b,c). The putative "gt" donor site formation, in addition to the predicted constitutive acceptor "ag" (located 38 base pairs upstream of the mutation site), may introduce a new exon codifying for 11 amino acids, due to the following “TGA” Stop codon (Fig. 3c).

The predictive analysis performed with DOMINO described this gene as having an autosomal dominant (AD) inheritance pattern (probability of AD: 0.6318). Furthermore, the variant was classified by FATHMM, Reg-SNP-intron and Mutation taster algorithms as disease causing (Table 1).

**Table 1:** In silico prediction of the pathogenic significance related to the variant c.1411-3C > T within *ARIH2* gene (NM_006321).

*Tool*	*Score *^*(a)*^	*Prediction*
FATHMM	0.972	Disease causing
RegSNPs-intron	0.64	Pathogenic supporting
Mutation taster	1.00	Disease causing

^***(a)***^According with the tools employed, scores spanned from 0 (benign) to 1 (disease causing).

*In-silico* splicing prediction analysis conducted using ASSP and Spliceator algorithms revealed that the identified variant created a novel “gt” donor gain site, as result of the genetic variant c.1411-3C > T within *ARIH2* (Fig. 3), further to the constitutive acceptor sites "ag", located 38 base pairs upstream of the mutation site. Both utilized tools categorized the variant site as a potential new alternative isoform or cryptic donor (Fig. 3 and Table 2). The newly generated donor site as a consequence of the mutation exhibits scores of 4.554 and 0.711 for ASSP and Spliceator, respectively. In addition, the prediction indicated the presence of a plausible constitutive acceptor site "ag" located 38 base pairs upstream of the mutation site exhibiting high scores (9.280 and higher than 0.7 according to ASSP and Spliceator, respectively) (Table 2). As predicted, both acceptor and donor sites may introduce a new exon consisting of 11 amino acids and a “TGA” Stop codon (Fig. 3).

**Table 2:** In silico splicing predictions for wild type *ARIH2* sequence and the c.1411-3C > T variation.

DNA variant	*Putative splice site*	*Position* ^(a)^	*Sequence* ^(b)^	*ASSP score*^(c)^	*Spliceator score*^(c)^
c.1411-3C (wild type)	Constitutive acceptor ^(d)^	48,983,149	ttgctccc**ag**GCCTGGGCCA	9.280	0.833
	Real acceptor^(e)^	48,983,190	tctgatgc**ag**GACTTGGAGA	7.960	0.701
c.1411-3C > T	Constitutive acceptor^(d)^	48,983,149	ttgctccc**ag**GCCTGGGCCA	9.280	0.787
	Alt. cryptic donor^(f)^	48,983,183	TTTCTCTGAt**gt**aggacttg	4.554	0.711
	Real acceptor^(e)^	48,983,190	tctgatgc**ag**GACTTGGAGA	7.960	0.701

^(a)^Position of the first nucleotide in the sequence shown in the next column of this table.

^(b)^Nucleotide sequences around the splicing site considered (indicated in bold). Uppercase letters indicate nucleotides in the exon region.

^(c)^Algorithms used for the prediction.

^(d)^Acceptor splicing site constitutively presents in the intron at the indicated position.

^(e)^Acceptor splicing site used in the wild type ARIH2 RNA processing.

^(f)^Alternative donor splicing site ”gt” generated by the c.1411-3C > T variant.

Significant letters are in bold.

In addition, the analyses conducted using NNSplice tool and Mutation Taster, revealed a candidate donor site alteration in the intronic region adjacent to the last exon of the *ARIH2* gene resulting with a splice site gain score of 0.37 (Table 3). Furthermore, PhyloP score exhibited the value of 1.58, indicating a moderate grade of conservation of the analysed splicing region. Conversely, PhastCons score for the splicing region at the mutation site was 0.914 (Table 3).

**Table 3:** Variation of the scores related to the inheritance and conservation of the *ARIH2* gene.

*Variation*	*Splice site gain score *^*(a)*^	*PhyloP*^*(b)*^	*PhastCons*^*(c)*^
(NM_006321) c.1411-3C > T^***(d)***^	0.37	1.58	0.914

^***(a)***^ Splice site gain score higher than 0.3 indicates gain of a completely new splice site. ^***(b)***^ PhyloP rate: spanning from -14 to 6. ^***(c)***^ PhastCons: ranging from 0 to 1. ^***(d)***^ Clinical variant n. SUB14199340 submitted on 14–02-2024 to ClinVar Portal NCBI. Variation ID: 2,692,232 Accession: VCV002692232.1.

### Hydrogen bonds assessment

**Figure 4:**
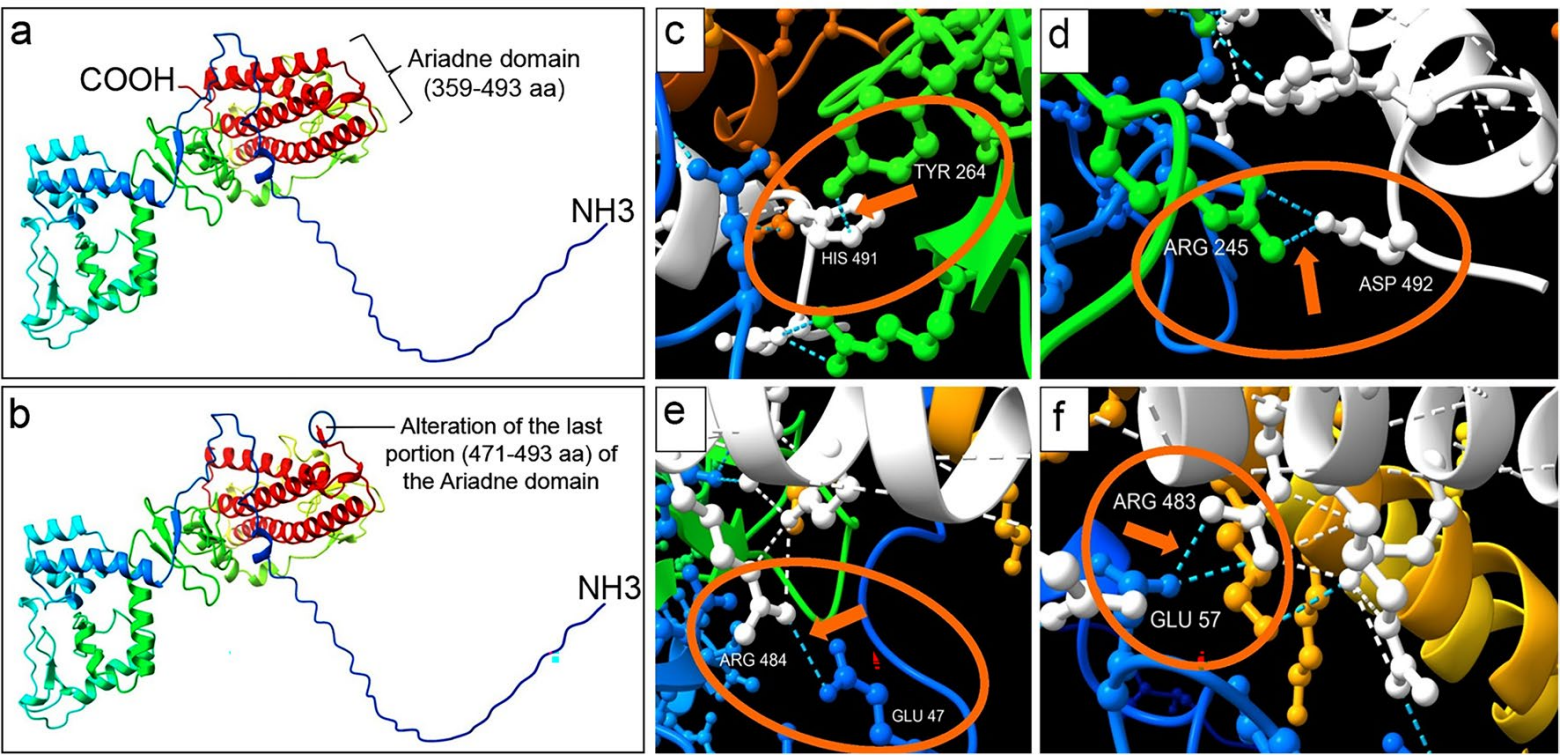
(**a**) Wild type and (**b**) mutated E3 ubiquitin protein ligase of *ARIH2* gene. (**c**-**f**) Ariadne domain: graphical representation of amino acid residues involved in hydrogen bond interactions in the last portion of the Ariadne domain (from 471 to 493 aa), including the last helix of the Ariadne domain (comprising from aa 469 to aa 491). This gene was affected by a de novo mutation in the intronic region at − 3 bp from the exon 16 of *ARIH2* gene. The figure illustrates six hydrogen bonds involving the region spanning from 471 to 493 aa and extra Ariadne domain residues: (**c**) one hydrogen bond between residues HIS491 and TYR264 (zinc finger RING 1 domain); (**d**) two hydrogen bonds between residues ASP492 and ARG245 (zinc finger RING 1 domain); (**e**) one hydrogen bond between residues ARG484 and GLU47 (UBA-like domain); (**f**) two hydrogen bonds between residues ARG483 and GLU57. The images were realized using the software UCSF Chimera X.

The analysis conducted using UCSF ChimeraX revealed a total of 42 hydrogen bonds within the region of the Ariadne domain, specifically corresponding to the segment affected by the mutation spanning from amino acid 471 to 493. These electrostatic bonds spanned between intra and extra Ariadne domain (359–493 aa). Notably, six of these interactions involved domains other than the Ariadne one (Fig. 4). Specifically, these bonds included interactions between the residues His491 (one hydrogen bond) and Asp492 (two hydrogen bonds) with Tyr264 and Arg245, respectively. Remarkably, both residues were situated within the zinc finger RING 1 domain. Additionally, these electrostatic interactions implicated the residues Arg483 (two hydrogen bonds) and Arg484 (one hydrogen bond), with interactions occurring between Glu57 (comprising an extra domain region) and Glu47 (UBA-like domain), respectively. STRING analysis revealed the correlation between E3 ubiquitin protein ligase and NEDD8, CUL5, RFN7 and UBE2L3 proteins. Specifically, these correlations were based on experimental evidence.

## Discussion

Genetic variants outside the canonical splicing sites can disrupt normal mRNA splicing, potentially impacting neurodevelopmental disorders clinically. Furthermore, mutations in genes involved in the ubiquitination process are often associated with ASD and/or ID^37,38^. The UPS intricately interplays with the non-lysosomal proteolytic pathway regulating cellular protein levels. This regulatory mechanism extends to proteins crucial for neuronal growth and function, emphasizing UPS's central role in modulating key cellular processes within the nervous system^12^.

Here we report details on the identification of a de novo, heterozygous mutation (c.1411-3C > T) localized in the splicing region (at − 3 bp from the last exon) in *ARIH2* gene, detected through next generation sequencing. This variation was detected in a patient exhibiting both ASD and ID. To date, *ARIH2* doesn’t have an assigned OMIM number and code. Nevertheless, the predictive analysis performed with DOMINO identified *ARIH2* gene as having an autosomal dominant (AD) inheritance pattern (probability of AD: 0.6318). The comprehensive whole exome analysis revealed no additional mutations directly or potentially associated with other known ASD and ID related genes. However, we cannot exclude the possibility that other genetic factors, undetectable by WES, may contribute to the phenotype. This specific variant has never been described in gnomAD and 1000G databases. We suggest a potential relationship between the pathogenic effect of this specific variant, based on the assessment conducted using the ACMG criteria. Additionally, the in silico analysis conducted using the Mutation Taster, FATHMM, and RegSNPs-intron tools revealed a potential pathogenic significance of the identified variant (Table 1).

Moreover, in silico splicing predictions conducted using the NNSplice, ASSP, and Spliceator algorithms revealed with high score that the identified variant (c.1411-3C > T) created a novel donor gain site, represented by the mutated "GT" sequence. In addition, the prediction analysis performed, indicated the presence of a plausible constitutive acceptor site "AG" located 38 base pairs upstream of the mutation site exhibiting high scores (Fig. 3, and Table 2). As predicted both acceptor and donor sites may introduce a new exon. This exon consists of 11 amino acids and a “TGA” stop codon.

For further support these findings, Mutation Taster yielded positive scores for both PhyloP and PhastCons, indicating a moderate and high conservation rate, respectively, at the specific site of the variant. Indeed, "GT" is a well-established sequence often associated with alternative donor sites in eukaryotic splicing^39^. Based on this prediction, we hypothesize that the splicing process alteration may disrupt the encoding of the last protein portion, resulting in a misfolded E3 ubiquitin protein ligase. Furthermore, it's important to note that intronic variants at position -3 in genes like *NF1*, *TSC1*, and *PAH*, (as indicated by HGMD database) constitute approximately 4% of causative mutations within intronic regions. This underscores the significance of exploring such variants in elucidating their potential contributions to pathogenicity, despite the limitations related to the possible presence of other variants not identified by WES analysis. We emphasize that a comprehensive understanding of the alternative splicing process would necessitate experimental studies on mRNA/cDNA, which, regrettably, are not currently possible.

Our hypothesis emphasizes that this splicing mutation alters the polypeptide chain of E3 ubiquitin protein ligase enzyme altering the last portion of the Ariadne domain (Fig. 2 and Fig. 4). The prediction of hydrogen bonds in the disrupted portion of the ARIH2 protein revealed the engagement of six significant hydrogen bonds between this region (471–493 aa) and other functional domains than Ariadne. Based on these findings, we propose that the absence of these electrostatic interactions has an impact on protein function. We underscore that the protein structure prediction conducted with UCSF ChimeraX considers elements such as the arrangement of alpha helices, beta strands, and loops. However, as limiting factor, it may not provide precise explicit modeling of metal ion binding sites like Zn^2+^. Nonetheless, as the prediction leverages the AlphaFold deep learning algorithm, it might indirectly capture certain metal ion interactions.

As previously documented, E3 ubiquitin protein ligase is autoinhibited by the Ariadne domain, which masks the active site within the second RING-type zinc finger domain, thereby inhibiting E3 activity^36^. Ariadne domain in Drosophila and *Caenorhabditis elegans* within *ARIH1* gene (paralog of *ARIH2*) have been implicated in the transcriptional and translational regulation^34,35^. As previously reported^40^, these processes, in a diverse group of genes, were associated with both syndromic and non‐syndromic forms of ASD. Despite *ARIH1* and *ARIH2* deriving from a common ancestral gene, their protein sequences exhibit low homology (34.91% overall and 38.06% in the Ariadne domain). Nevertheless, they have the same domains pattern organization (AC-UBA-RING1-IBR-RING2-Ariadne). While limited structural information exists on the IBR domain, protein structure predictions suggest potential hydrogen bond formation between the IBR and Ariadne domains (specifically between Asp 492 and Arg 245, as well for His 491 and Tyr 264) (Fig. 4). Given the structural proximity identified between the Ariadne and IBR domains, these electrostatic interactions seem plausible based on our protein structure prediction analysis^41–43^. Building upon these hypotheses, it is likely that the disruption of the Ariadne domain significantly impacts, altering post-transcriptional modifications of the target protein, which might potentially contribute to the development of ASD and ID.

As previously documented, E3 ligases genes have been linked to neurological disorders that include neurodevelopmental or neurodegenerative diseases, such as autism, schizophrenia, intellectual disability, epilepsy, Angelman syndrome, and Parkinson’s disease^9,13,17–20^. As was outlined, the alteration of the E3 ubiquitin protein ligase structure in patients showing ASD, negatively interacts with synaptic function and plasticity, altering learning and memory formation by targeting activity regulated cytoskeleton-associated protein (Arc), a Rho guanine nucleotide exchange factor (Ephexin5), and small conductance calcium-activated potassium channel (SK2)^37,44,45^. In particular, several E3 ligases are encoded by various genes, such as *UBE3A*, *UBE3B*, *TRIP12* and *HUWE1* showing specific pathogenic variations associated to Autism^37,38,46^.

Interestingly, *ARIH2* gene expression was observed in several brain tissues, including amygdala, basal ganglion, cerebellar hemispheres, cerebellar vermis and cerebral cortex, tissues potentially involved in ASD^30–32,47^. Moreover, previous studies indicated a differential expression of *ARIH2* in post-mortem cerebral cortex tissue between individuals with ASD and neurotypical controls. Specifically, *ARIH2* was found to be downregulated in ASD patients^48,49^.

*ARIH2* was investigated in a cohort of patients analyzed for the detection of rare coding variations by population genetic approach, aiming to provide valuable insights into the genetic architecture and phenotypic context of autism^50^. Within this context, two de novo variants were detected in 5’ UTR and intronic regions of *ARIH2* gene, exhibiting an uncertain correlation with autism. It’s worth mentioning that an additional de novo mutation, in the *ARIH2* gene, involving a repeated motif AAG-1 was previously reported in a proband affected by ASD^51^. A prior study conducted a predictive analysis focused on loss-of-function mutations in six genes, considering factors such as the extent of brain expression, haploinsufficiency index, functional knowledge about gene homology, and insights from mouse models. This analysis identified *ARIH2* as a candidate gene associated with ID^52^. Furthermore, *ARIH2* was postulated to be part of a subset of intelligence-related genes, characterized by intra-species variations in human populations. These genes may also be evolution-related, exhibiting inter-species variations between humans (*Homo sapiens*) and great apes (including *P. troglodytes* and *P. abelii*)^53^.

The analysis performed with STRING based on experimental evidence of the protein pathways through the study of protein–protein interactions, revealed a significant allosteric specificity among ARIH2, CUL5 and NEDD8 genes. The latter was reported as likely associated to ASD^45^. Specifically, all these genes (*ARIH2*, *NEDD8*, *CUL5*) interplay with the conjugation and ubiquitination activities mediating the subsequent proteasomal degradation of target proteins (Fig. 2b). Notably, *NEDD8* uniquely contacts covalently linked *CUL5*, inducing structural rearrangements that expose previously hidden regions of *ARIH2*^26^. Furthermore, there is evidence supporting the role of NEDD8 and CUL5 in regulating mammalian excitatory synapses and neural development^54,55^.

Notably, we are hypothesizing that the splicing mutation that we detected might be associated with ASD and ID similarly to the prior studied mutations in genes encoding for E3 ubiquitin protein ligase. Additionally, it is important to emphasize a potential limitation: variants in non-coding regions, such as those associated with promoter regions, may also contribute to the ASD phenotype^56^. Within this context, it is essential to acknowledge that while Whole Exome Sequencing (WES) is a powerful tool for identifying coding mutations, it may not capture genetic variations in non-coding regions or complex polygenic interactions^1,57^. These undetected mutations, including those in non-coding regions and polygenic risks arising from inter-allelic complementation, could potentially play significant roles in shaping the patient's phenotype^58^. A limitation of our WES analysis is the filtering out of polymorphisms with Minor Allele Frequencies (MAF) higher than 1%, which may contribute to polygenic risk. The limitations of WES in detecting these variants underscore the need for complementary approaches, such as Whole Genome Sequencing (WGS) or targeted sequencing of specific regulatory elements, to fully elucidate the genetic landscape underlying complex neurodevelopmental disorders^59,60^. Integrating these techniques with advanced bioinformatic analyses will be crucial for uncovering the complete spectrum of genetic factors contributing to the phenotype.

Although several studies have identified the strong role of ubiquitination in autism and intellectual disability, the association between *ARIH2* gene and these conditions that we are proposing is only a hypothesis. In fact, further patients exhibiting ASD or ID with variants in the *ARIH2* gene are needed to validate our study. Further in vitro functional assays on *ARIH2* gene are essential to test the validity of the novel gene and to enhance our understanding of the complex interactions among genes encoding proteins in ASD.

Understanding ubiquitination's role in cellular processes offers insight into disease mechanisms and therapeutic avenues. Drugs targeting ubiquitin enzymes can modulate protein degradation, suggesting potential therapeutic interventions^61–63^. Therefore, comprehending ubiquitination and its implications in cellular physiology and pathology can lay the basis for the development of novel therapeutic approaches for various diseases.

## Conclusion

In this study, we report a patient showing both ASD and ID. Whole exome sequencing (WES) enabled the identification of a de novo heterozygous mutation in the *ARIH2* gene encoding for an E3 ubiquitin protein ligase. Notably, this novel mutation is located in the intronic region adjacent to the last exon of this gene. Based on a comprehensive assessment of its plausible pathogenic role, we hypothesize a potential relationship between ARIH2 and the patient's phenotype. Nevertheless, we cannot rule out the possibility that other genetic factors, which are undetectable by WES, could determinate or contribute to the patient's phenotype. It is worth mentioning that E3 ubiquitin ligases are a large family of enzymes widely documented as implicated in ASD and ID. Our hypothesis is that the mutation alters the last portion of the Ariadne domain involved in autoinhibition of the E3 ubiquitin ligase protein modulating proteasomal degradation. *ARIH2* gene lacks an assigned OMIM number. We propose for the first time an association between this gene and ASD/ID. Further functional studies are needed in autistic patients exhibiting ARIH2 variants, to validate the current study.

## Materials and methods

### Library preparation and NGS analysis

Genomic DNA was isolated from peripheral blood leukocytes obtained from the clinical case, as well from the father and the mother. The extraction protocol applied was a non-organic and non-enzymatic extraction method previously developed^64^. Exome analysis was performed using the Ion AmpliSeq™ Exome RDY kits, following the manufacturer’s instructions (Thermo Fisher Scientific, Waltham, MA, USA). The quality of libraries was assessed using DNA 1000 chips on the Tape Station 4200 (Agilent, Santa Clara, CA, USA) and Qubit dsDNA BR Assay kits (Invitrogen, Waltham, MA, USA). Template preparation, clonal amplification, recovery, and enrichment of template-positive Ion Sphere™ particles and loading of sequencing-ready Ion Torrent semiconductor chips were performed with the Ion Chef™ system (Thermo Fisher Scientific, Waltham, MA, USA). Finally, we sequenced each loaded Ion 550™ chip on the S5 system (Thermo Fisher Scientific, Waltham, MA, USA). Overall, 98% of regions of interest have a minimum coverage of at least 20 × . Data of runs were processed using the Ion Torrent Suite 5.16, Variant Caller 5.16, Coverage Analysis 5.16 (Thermo Fisher Scientific, Waltham, MA, USA), Ion Reporter (Thermo Fisher Scientific, Waltham, MA, USA), and/or wANNOVAR tools^65^. DNA sequences were displayed using Integrated Genomics Viewer^66^. The reference genome employed for the analysis was Hg38. Pathogenic variant was confirmed through conventional Sanger sequencing. The methods related to the Variant Caller parameters and PCR setup were available in the Supplementary File 1. DNA fingerprint analysis was performed for both patient and parents, according to a previous protocol^67^, to confirm maternity and paternity, and de novo event.

### Data analysis

The observed nucleotide variation was classified according to the “American College of Medical Genetics” (ACMG) guidelines^68^ and it was performed with VarSome^69^, and the three criteria used for the classification used were reported in Table 4.

**Table 4:** Criteria used for the classification of the observed nucleotide variation.

Criteria for classifying variants ^(a)^	Category code	Description
Strong	PS2	De novo (both maternity and paternity confirmed) in a patient with the disease and no family history
Moderate	PM2	Absent from controls (or at extremely low frequency if recessive) in Exome Sequencing Project, 1000 Genomes Project, or Exome Aggregation Consortium
Supporting	PP4	Patient’s phenotype or family history is highly specific for a disease with a single genetic etiology
ACMG variant classification		Likely Pathogenic

^(a)^According to the American College of Medical Genetics (ACMG) guidelines^56^**.**

For the in silico evaluation of the pathogenic significance of the variant, we utilized the MutationTaster algorithm (https://www.mutationtaster.org/) (accessed on 15 November 2023) along with the FATHMM (https://fathmm.biocompute.org.uk/) and RegSNPs-intron (https://regsnps-intron.ccbb.iupui.edu/) tools (accessed on 17 April 2024). These tools assign output values ranging from 0 to 1, indicating the pathogenic significance of the variant^70,71^.

Protein sequence and domains were obtained from the free online database of UniProt (https://www.uniprot.org/) (accessed on 15 November 2023) (UniProt Consortium, 2015). Expression level was ascertained on The Human Protein Atlas (https://www.proteinatlas.org/) (accessed on 15 November 2023)^72^.

The in silico prediction of the splicing process was performed using NNSplice tool, provided by Mutation Taster website, in addition to ASSP (http://wangcomputing.com/assp/) and Spliceator (https://www.lbgi.fr/spliceator/) tools (accessed on 17 April 2024). ASSP scores range from 0 to 10, with higher scores indicating increased likelihood of a splice site. Cutoff values of 2.2 for acceptor sites and 4.5 for donor sites achieve correct identification rates of approximately 75% and 80%, respectively, for false and alternative isoform/cryptic splice sites^73,74^. Spliceator scores range from 0 to 1, with higher scores indicating higher likelihood of a sequence being recognized as a splice site^75^.

The confidence, PhyloP and PhastCons scores were analysed according to previous studies^76–78^. Notably, a mutation taster splice site gain score higher than 0.3 indicates gain of a completely new splice site. Conversely, PhyloP and PhastCons scores assess conservation rate in the analysed region. Within this context, PhyloP score spans between -14 and 6. Sites predicted to be conserved are assigned positive scores, while sites predicted to be fast evolving are assigned negative scores. On the other hand, PhastCons range between 0 and 1.

Additionally, in silico conservation rate was analyzed by DOMINO with the score provided by VarSome database (accessed on 15 November 2023)^69^, according to prior research^79,80^. For all the bioinformatic tools employed were used the default parameters. The Human Gene Mutation Database (HGMD) (https://www.hgmd.cf.ac.uk/) (accessed on 17 April 2024)^81^ was utilized to determine the percentage of intronic variants located at position -3 from various exons, which have been confirmed to have causative effects in genes such as NF1, TSC1, and PAH. STRING analysis was conducted for analyzing the protein pathways correlated with *ARIH2* gene based on experimental studies (https://string-db.org/) (accessed on 15 November 2023)^82^.

Molecular graphics and analyses performed with UCSF ChimeraX, developed by the Resource for Biocomputing, Visualization, and Informatics at the University of California, San Francisco, with support from National Institutes of Health R01-GM129325 and the Office of Cyber Infrastructure and Computational Biology, National Institute of Allergy and Infectious Diseases^83^.

### Institutional review board statement

All procedures performed in this study were in accordance with the ethical standards of the institutional and/or national research committee and with the 1964 Helsinki Declaration and its later amendments or comparable ethical standards. This study was conducted in accordance with the Declaration of Helsinki and approved by the local Ethics Committee “Comitato Etico IRCCS Sicilia-Oasi Maria SS”. Prot. CE/88bis, as of 20 March 2019, approval code: 2019/03/18/CE-IRCCS-OASI/18.

### Informed consent statement

Written informed consent has been obtained from the patient’s parents to publish this article.

### Supplementary Information

Supplementary Information.

## Data Availability

The datasets analysed during the current study are available in the ClinVar repository, acc. No. VCV002692232.1 (Variation ID: 2692232). [Web link: https://www.ncbi.nlm.nih.gov/clinvar/variation/2692232/?oq=2692232&m=NM_006321.4(ARIH2):c.1411-3C%3ET]. Figure 2C data are from Uniprot protein database. Accession: https://www.uniprot.org/uniprotkb/O95376/entry, family & domains section. Accessed on 19/02/2024.
