# Supplementary material for: A de novo ARIH2 gene mutation was detected in a patient with autism spectrum disorders and intellectual disability
Source: Sci Rep. 2024 Jul 9;14:15848. doi: 10.1038/s41598-024-66475-2 (PMC11233510; doi:10.1038/s41598-024-66475-2)
Supplement: Supplementary file 1 — Supplementary Information. [file 41598_2024_66475_MOESM1_ESM.docx]

**Supplementary file-1:** Variant calling parameters and PCR setup conditions

**
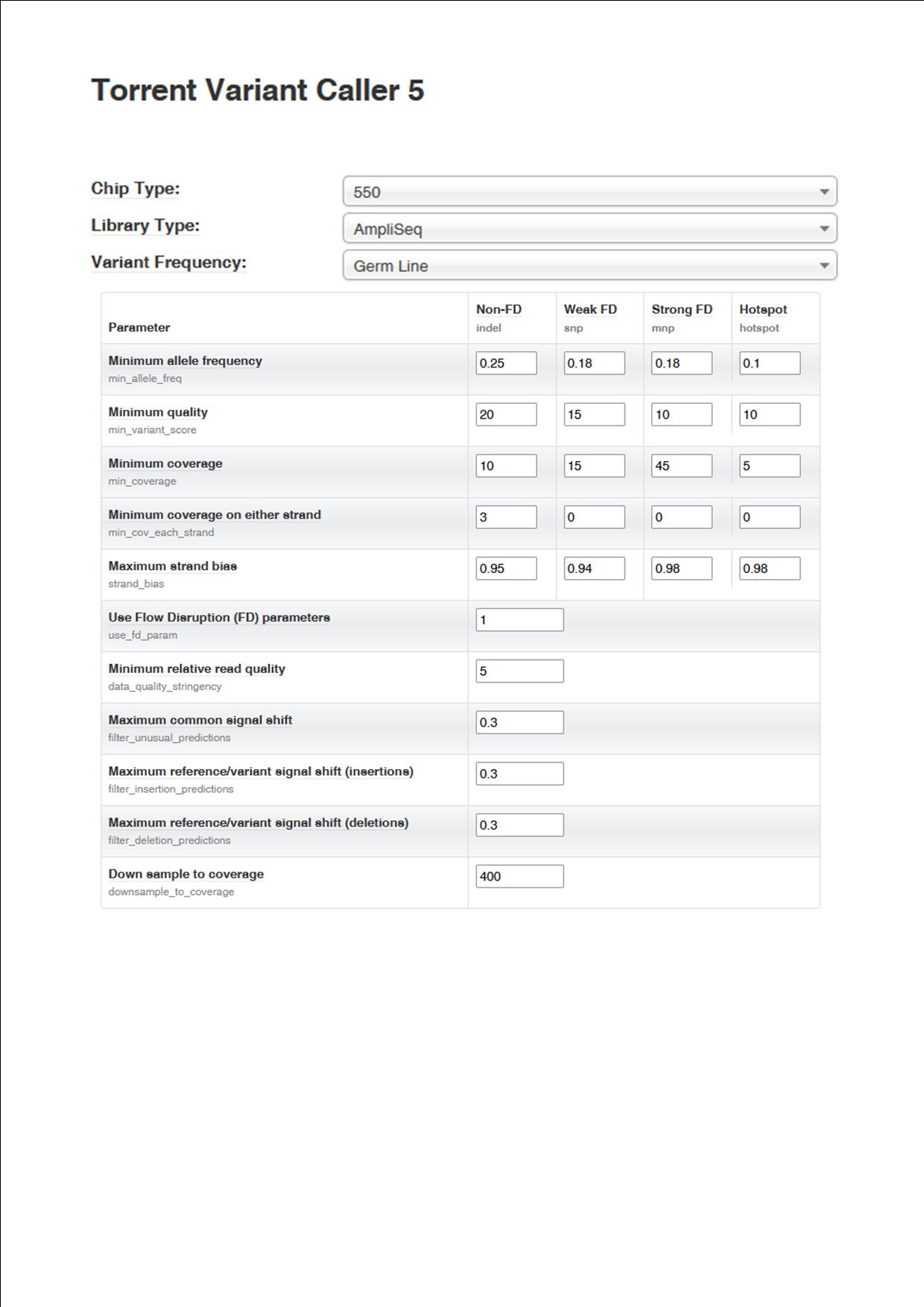
**

**Variant caller parameters**

**Figure S1.** **Variant caller parameters.** Data of runs were processed using the Ion Torrent Suite 5.16 and Variant Caller 5.16 (Thermo Fisher Scientific, Waltham, MA, USA), Ion Reporter (Thermo Fisher Scientific, Waltham, MA, USA).

**PCR setup conditions**

The primers flanking the target region were generated employing the specific National Center for Biotechnology Information (NCBI) web tool (<https://www.ncbi.nlm.nih.gov/tools/primer-blast/>) and presented in **Table S1**.

**Table S1.** Sequences flanking the target ARIH2 region related used as primer for the conventional Sanger sequencing, The sequences were developed by primers design tool available from NCBI website (<https://www.ncbi.nlm.nih.gov/tools/primer-blast/>).

| **Gene** | **Primer forward** | **Primer reverse** | **Tm (°C)** |
| --- | --- | --- | --- |
| ARIH2 | CTGGAGGGTGTTTGTGGCT | GGCACATCCACATCCCAACT | 59.4 |

PCR was performed for the patient and both parents in 50 μl per reaction. DNA concentration was 30-40 ng μl­^-1­^, while primers were used at the concentration of 15 pmol μl­^-1^. Additionally, 5u of GoTaq® G2 DNA Polymerase (Promega Corporation, Fitchburg, MA, USA) were included in the reaction. The amplification protocol included an initial denaturation step at 94 °C for six minutes, followed by denaturation at 94 °C for 30 seconds, annealing at the melting temperature for 30 seconds, and extension at 72 °C for 1 minute. This pattern was repeated for 35 cycles, with a final extension step at 72 °C for 10 minutes, followed by a final hold at 4 °C. Subsequently, the first purification step was performed using 5 μl of amplicon and 1.5 μl of GenUP™ Exo SAP Kit (Biotech rabbitGmbH, Berlin, Germany) for 15 minutes. Sequence amplification was carried out using the BigDye™ Terminator v1.1 Cycle Sequencing Kit (ThermoFisher Scientific, Waltham, MA, USA). The final purification step was conducted using the DyeEx 2.0 Spin Kit (Qiagen, Venlo, Netherlands), and Sanger sequencing was performed on the Applied Biosystems Prism 3130 DNA Analyzer following the manufacturer’s instructions (ThermoFisher Scientific, Waltham, MA, USA).
